# EEG-Based Target Detection Using an RSVP Paradigm under Five Levels of Weak Hidden Conditions

**DOI:** 10.3390/brainsci13111583

**Published:** 2023-11-12

**Authors:** Jinling Lian, Xin Qiao, Yuwei Zhao, Siwei Li, Changyong Wang, Jin Zhou

**Affiliations:** 1 Department of Neural Engineering and Biological Interdisciplinary Studies, Beijing Institute of Basic Medical Sciences, 27 Taiping Rd., Beijing 100850, China; lianjinling@bit.edu.cn (J.L.); qiaoxinsdu@126.com (X.Q.); zhaoyuwei158@163.com (Y.Z.); lsw2005010608@163.com (S.L.); 2 Chinese Institute for Brain Research, Zhongguancun Life Science Park, Changping District, Beijing 102206, China

**Keywords:** electroencephalogram, event-related potential, rapid serial visual presentation, target detection, weak hidden conditions

## Abstract

Although target detection based on electroencephalogram (EEG) signals has been extensively investigated recently, EEG-based target detection under weak hidden conditions remains a problem. In this paper, we proposed a rapid serial visual presentation (RSVP) paradigm for target detection corresponding to five levels of weak hidden conditions quantitively based on the RGB color space. Eighteen subjects participated in the experiment, and the neural signatures, including P300 amplitude and latency, were investigated. Detection performance was evaluated under five levels of weak hidden conditions using the linear discrimination analysis and support vector machine classifiers on different channel sets. The experimental results showed that, compared with the benchmark condition, (1) the P300 amplitude significantly decreased (8.92 ± 1.24 μV versus 7.84 ± 1.40 μV, *p* = 0.021) and latency was significantly prolonged (582.39 ± 25.02 ms versus 643.83 ± 26.16 ms, *p* = 0.028) only under the weakest hidden condition, and (2) the detection accuracy decreased by less than 2% (75.04 ± 3.24% versus 73.35 ± 3.15%, *p* = 0.029) with a more than 90% reduction in channel number (62 channels versus 6 channels), determined using the proposed channel selection method under the weakest hidden condition. Our study can provide new insights into target detection under weak hidden conditions based on EEG signals with a rapid serial visual presentation paradigm. In addition, it may expand the application of brain–computer interfaces in EEG-based target detection areas.

## 1. Introduction

The brain–computer interface (BCI) can build a direct communication link between humans and the outside world by translating complex, massive, and nonstationary brain signals into interaction commands. This can provide an alternative or additional way for human–machine interactions to take place [1]. Recently, due to the low cost, convenient usability, non-invasiveness, and high time resolution of electroencephalogram (EEG) recording, brain–computer interfaces (BCIs) based on EEG have been widely utilized to explore brain-controlled applications. For disabled people, these applications include spellers [2,3], robotic arms [4,5], robots [6,7], vehicles [8,9], and unmanned aerial vehicles (UAVs) [10]. These were developed in the disability assistance field to facilitate communication with external devices. For non-disabled people, these applications include secondary task assistant systems [11,12], third arms [13], emotion recognition [14,15], concentration evaluation [16], drowsiness detection [17], and target detection [18]. These were developed to enhance the efficiency of operators.

Among the various BCI applications, BCI based on rapid serial visual presentation (RSVP) is a typical BCI for target detection. It can present an image stream and detect the presence or absence of any interesting targets (e.g., text, a number, a human, a vehicle, or an airplane) by collecting and analyzing EEG signals during the presentation. The purpose of the RSVP-based BCI is to detect a target through the neural signature of the brain pattern instead of a delayed behavioral response. The target of interest will induce the event-related potentials (ERP) in the EEG signals while a non-target will not. The EEG signals corresponding to a target of interest and a non-target can be collected and analyzed. Then, new EEG signals corresponding to a picture can be classified into a target category or non-target category using machine learning methods. We then can infer whether this picture contains a target or not. The existing applications of RSVP-based BCI include surveillance [19,20], face recognition [21,22], medical image analysis [23], and RSVP spellers [24,25,26]. Due to its relatively high detection speed compared with manual operation, especially for detecting targets from multiple huge images with high resolution, RSVP-based BCI is considered to be a potential approach for enhancing the ability and improving the efficiency of operators [27,28,29,30,31].

Recently, there have been numerous studies [27,28,29,30,31] using the RSVP-based BCI for target detection. Manor et al. proposed an RSVP paradigm for detecting various kinds of structures like buildings or roads [27]. These structures were artificially considered as one type of target in this study. The images not containing these structures but containing various patterns of ground plants or other natural items were considered non-targets. Fernandez and Poli proposed an RSVP paradigm to detect a randomly rotated, positioned, and superimposed airplane from aerial pictures of London [28], in which target localization was also investigated. Wei et al. proposed an RSVP paradigm to detect pedestrians [29], in which one or more pedestrians were considered as one type of target and the images of the street scenes without pedestrians were considered to be non-targets, from the database of the Massachusetts Institute of Technology, Computer Science and Artificial Intelligence Laboratory (MIT-CSAIL). Marathe et al. chose moving or static persons and vehicles as one type of target and open country background scenes as non-targets when proposing an RSVP paradigm, which was presented with short video clips [30]. Unlike studies with a single type of target, Li et al. proposed and improved an RSVP paradigm containing two types of targets, including a human face and table [31], and various natural scenery pictures as non-targets. Although the above studies focused on the detection of the salient (e.g., conspicuous, outstanding, and brilliant) targets (single type or two types) from the various interference non-targets, the detection of targets under weak hidden conditions using the RSVP paradigm also has considerable value in medical applications. For medical applications, a higher detection performance to detect a target under weak hidden conditions will make for a lower missing error rate and the timely diagnosis of diseases. For other applications, target detection and searching under weak hidden conditions is helpful in the early identification of indistinct targets (e.g., humans, ground carriers and vehicles, small UAVs in a concealed environment for aerial images, and manned aircraft in satellite images).

Fan et al. proposed a paradigm with a slower image presentation speed called the asynchronous visual evoked paradigm (AVEP) to detect a dim target (airplane) in satellite images [32]. Considering the unpredictable willingness of the subject, the asynchronous function was added to detect the dim target with a long period of paradigm presentation time. Due to the low presentation speed, this study’s paradigm cannot technically be called an RSVP paradigm. Moreover, “dim” was not defined, either qualitatively or quantitatively. As a result, the different levels of dim targets were not grouped and not investigated. To the best of our knowledge, no studies have focused on target detection under different levels of weak hidden conditions based on the RSVP paradigm using EEG signals. During the target detection, different levels of weak hidden conditions may lead to different EEG responses and neural signatures, probably resulting in different detection performances in practice.

In this study, we defined the weak hidden conditions quantitatively based on the RGB color space and then designed RSVP paradigms corresponding to five levels of weak hidden conditions. Stimuli for a high level of hidden conditions were set with a lower RGB value to make the stimuli more hidden, i.e., hard to recognize with human visual perception. Stimuli for a low level of hidden conditions were set with a higher RGB value to make the stimuli easier to recognize with human visual perception. The degrees of weak hidden conditions proposed in this study can be perceived qualitatively by human vision. But, a human cannot quantify the stimuli for different levels of weak hidden conditions. So, we quantitively defined the weak hidden conditions based on the RGB color space for this study. For other types of weak hidden conditions (mentioned in Section 4), it is possible that other quantitative indicators could be used to define these conditions. EEG signals from 62 channels were collected and neural signatures, including amplitude and latency, were explored for each paradigm under different weak hidden conditions. Then, the optimal channel sets were determined by the channel selection method for each subject under each weak hidden condition. Finally, detection performance, including the classification accuracy and information transfer rate, was evaluated and compared using the linear discrimination analysis and support vector machine classifiers under five levels of weak hidden conditions.

The major contributions of this study are as follows:

(1) Five paradigms were proposed, corresponding to five levels of weak hidden conditions, which were quantitatively defined based on the RGB color space;
(2) Neural signatures, including P300 amplitude and latency under the five levels of weak hidden conditions, were analyzed and compared statistically;
(3) A channel selection method was proposed and different channel sets were investigated to decode their performance.

Our study can provide a valuable reference and new insights into target detection under weak hidden conditions based on EEG signals with a rapid serial visual presentation paradigm. This may also expand the application of the brain–computer interface in the EEG-based target detection field.

## 2. Materials and Methods

**Figure 1:**
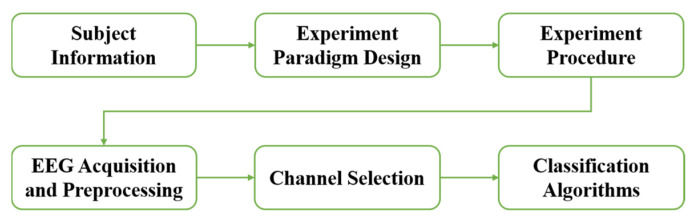
The block diagram presentation of the proposed experiment.

A block diagram presentation of the proposed experiment is shown in Figure 1. The details of each part of Figure 1 are described below in Section 2.1, Section 2.2, Section 2.3, Section 2.4, Section 2.5 and Section 2.6.

### 2.1. Subject Information

Eighteen subjects (11 males and 7 females, 26.11 ± 2.52 years) participated in the experiment. All subjects had normal or corrected-to-normal vision and had no neural-related diseases. The study adhered to the principles of and was conducted in accordance with the 2013 Declaration of Helsinki, and it was approved by the Ethics Committee of the Academy of Military Medical Sciences (protocol code AF/SC-08/02.309). All subjects signed the informed consent form after the experiment purpose, the required tasks, and the possible consequences were explained. Participants were paid for their participation.

### 2.2. Experiment Paradigm

We designed experimental paradigms for target detection under five levels of weak hidden conditions, with half-and-half from the benchmark condition (pure white with R: 255, G: 255, and B: 255) based on RGB color space. The reasons for quantifying the levels using RGB color space rather than other metrics were as follows: (1) RGB color space is considered to be the base color space for various applications [33], and it is the most widely used color model [34] and is closest to a nature scene [35]; (2) although the RGB color space can be transformed into a grey color space [36], the focal point of this study was not to investigate the grey stimuli and different RGB colors that can be transformed into the same grey color space; and (3) a comprehensive metric, e.g., root mean square clutter metric or probability-of-edge metric [37], was not adopted because similar metric values can be obtained by different stimuli, which may lead to different recognition performance. All five experimental paradigms were the same, except for the RGB of the stimuli number with a pure black background (R: 0, G: 0, B: 0). The RGB value of the stimuli number and background and the ratio between the value and 255 are shown in Table 1.

**Figure 2:**
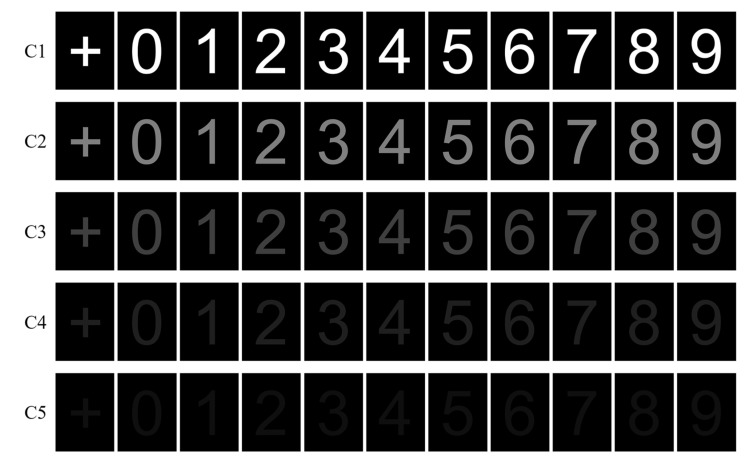
Stimuli for five RSVP paradigms corresponding to five levels of weak hidden conditions.

**Figure 3:**
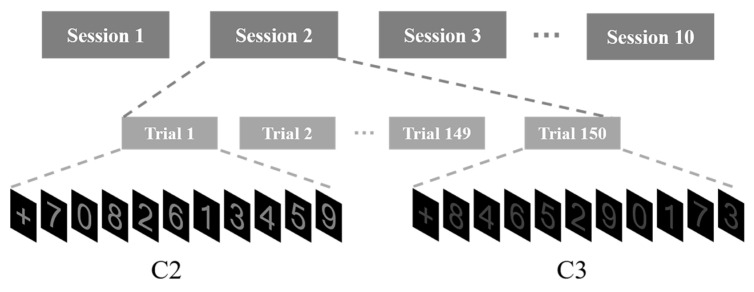
The experimental protocols.

The stimulus pictures used for each paradigm under different levels of weak hidden conditions are shown in Figure 2. In total, five paradigms corresponding to five conditions (C1, C2, C3, C4, and C5) were proposed. C1 was considered to be the benchmark condition because the stimuli under this condition are most conspicuous compared with other conditions. Each paradigm included ten numbers from 0 to 9 and a plus sign with the same RGB value as the numbers. In each trial, after the plus sign was presented for one second, followed by a 0.5 s gap, each number was presented for 200 milliseconds in a pseudo-random sequence with no inter-stimulus interval. The pictures had a size of 227 pixels × 302 pixels (approximately 3:4). The experiment included 10 sessions, and each session consisted of 150 trials (30 trials for each weak hidden condition) with a random sequence, as shown in Figure 3.

### 2.3. Experimental Procedure

After providing signed informed consent prior to the experiment, the subjects sat in front of a Samsung LED display (19 inches, refresh rate 60 Hz, resolution 1440 × 900) with a distance of approximately 50 cm. Because distance is considered to be an influencing factor, the subject was asked to keep the distance as fixed as possible during the experiments. The brightness and contrast ratio of the display was set to the maximum. The experimenter explained the entire experimental procedure to the subject and carried out experimental preparations. The impedances between the scalp and the electrodes at each channel position were adjusted under 10 KΩ. Before the formal experiment, the subjects were instructed and became familiar with the experimental paradigms. Once the target number (informed beforehand) appeared, the subjects were asked to react rapidly in their brain. During the formal experiment, when one session was completed, subjects could ask for a short break of several minutes to relax their eyes at their discretion.

### 2.4. EEG Acquisition and Preprocessing

Australian commercial EEG acquisition equipment, the NeuroScan SynAmps2 system (Compumedics Ltd., Melbourne, Australia), was used to acquire the EEG signals. The EEG signals were collected with a sampling rate of 1000 Hz and a channel number of 62 according to the 10–10 electrode system. The reference electrode was at the vertex. The EEG samples corresponding to each stimulus (target stimulus and non-target stimulus) were segmented from the onset of the stimulus to 1000 milliseconds post-stimulus for each paradigm. A total of 300 target samples and 2700 non-target samples were collected for each subject under every condition. The original signals were then band-pass filtered from 0.3 Hz to 20 Hz and down-sampled with a factor of 8.

### 2.5. Channel Selection

**Figure 4:**
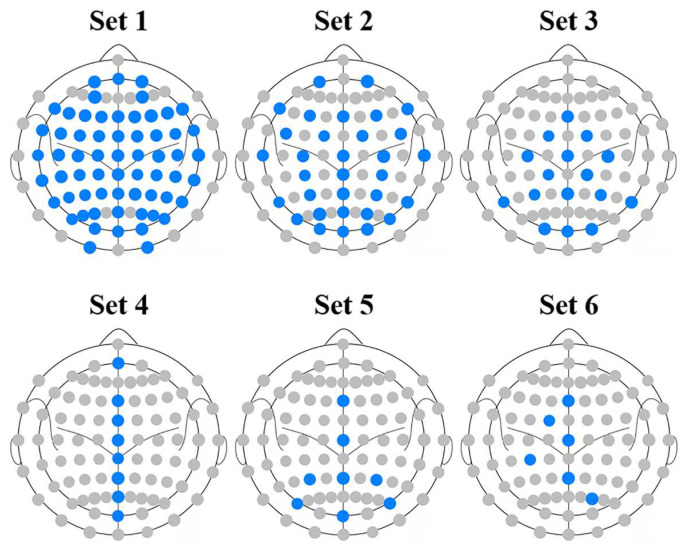
The channel layouts of different channel sets. The used channels are marked in blue disks. Note that channel set 6 is only an example, and the layout may be changed for different subjects and conditions.

Different channels at different locations provide various brain information, which may contribute to different levels of recognition performance. Fewer channels will make for a shorter system setup time and a lower cost, which are helpful for practical use. In this study, we investigated six different channel sets used for detecting recognition performance. Channel set 1 consisted of all channels, with a total number of 62. Channel set 2 and channel set 5 consisted of 32 and 8 channels, according to [38], respectively. Channel set 3 consisted of 16 channels, according to [39]. Channel set 4 consisted of 8 channels distributed on the centerline of the brain topography. Channel set 6 consisted of the channels selected by the further improved forward floating search algorithm using an adaptive principal component analysis based on our previous study [40]. More narrowly, before each period of calculating the within-class scatter matrix and the between-class scatter matrix, the current features were compressed to reduce the dimensionality by an adaptive principal component analysis to cover more than 99% of the information. We chose the first 6 channels from all of the selected optimal channel sets. Thus, channel set 6 only consisted of 6 channels for the different subjects under different conditions. Table 2 shows detailed information regarding the six channel sets. Figure 4 shows the channel layouts of different channel sets, with the used channels marked in the blue disks.

### 2.6. Classification Algorithms

For each channel set under different conditions, the original features for classification can be represented as $X\in {ℜ}^{N\times C}$. *N* denotes the sample points after the EEG signals were down-sampled, and *C* denotes the channel number of the channel set. After preprocessing, the original features were compressed, and the feature dimensionality was reduced using principal component analysis. The components with the highest *P* eigenvalues were chosen as feature weights and new features can be presented as $x={\left[x(1),\;x(2),\;\ldots ,\;x(i),\;\ldots ,x(P)\right]}^{T}$. *P* was adaptively determined by the contribution to more than 99% of the information of the original features for each subject under each condition. Then, the linear discrimination analysis (LDA) and support vector machine (SVM) classifiers were used for training the classification model. The classifier built by LDA can be represented as

(1) $$y=w^{T}x$$

where *w* represents the projection direction. The threshold ξ was determined by the receiver of the curve (ROC). If the score y was larger than ξ, the sample was classified into the target class; otherwise, the sample was classified into the non-target class. The classifier built by the SVM with radial basis function (RBF) as the kernel function can be represented as

(2) $$y=\sum_{i=1}^{n}w_{i}\exp\left(-g{\left\|x_{i}-x\right\|}^{2}\right)+b$$

where $x_{i}$ is the *i*th support vector (SV) of the classifier, $w_{i}$ is the weight of the *i*th SV of the classifier, *n* is the number of the SV of the classifier, *g* is the width of the RBF of the classifier, and *b* is the bias of the classifier. We used the LIBSVM software (Version 2.0) library proposed by Chang and Lin to train the parameters of the SVM classifier [41]. Finally, these models were evaluated by the test dataset. Furthermore, a 10 × 6-fold cross-validation strategy was used to eliminate the random distortion effectiveness by grouping samples.

The information transfer rate was calculated according to that in [3] using the following equations:

(3) $$ITR=\frac{60}{T}\left[\log_{2}N+P\log_{2}P+(1-P)\log_{2}\left(\frac{1-P}{N-1}\right)\right]$$

where *T* denotes the period for issuing one command, *N* represents the numbers of all commands, and *P* represents the recognition accuracy.

## 3. Results

### 3.1. ERP Wave Morphology under Five Levels of Weak Hidden Conditions

**Figure 5:**
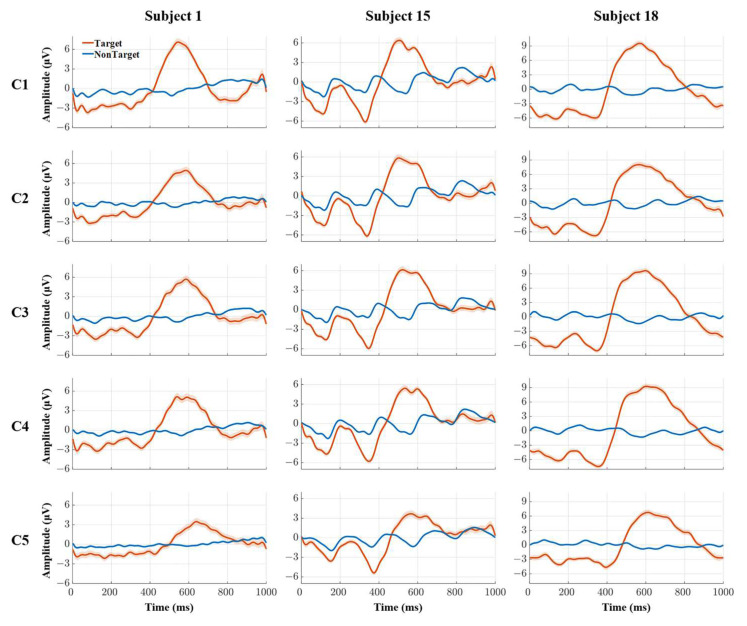
ERP wave morphology for subjects 1, 15, and 18 under five levels of weak hidden conditions.

EEG segments were extracted from the onset of the stimulus to 1000 ms post-stimulus. Figure 5 shows the ERP wave morphology at channel Fz elicited by targets and non-targets under five conditions for subject 1, subject 15, and subject 18. Channel Fz was investigated as the largest P300 amplitude was found at Channel Fz in our previous study [42]. The horizontal axis represents the time, and the vertical axis represents the amplitude of the EEG signals. The red line represents the ERP wave morphology corresponding to targets, and the blue line represents the EEG wave morphology corresponding to non-targets. The shadow around the lines represents the standard error corresponding to signal waves at specific time points. From Figure 5, we can see that the ERP was successfully elicited by targets for the subjects under five conditions, and the ERP wave morphology differed from the subject, as shown by the red lines. In contrast, no ERP wave morphology was found for non-targets under these conditions. The amplitude range of the targets differed from the subject. The amplitude of the ERP of subjects 1, 15, and 18 ranged from −4 μV to 7 μV, from −6 μV to 7 μV, and from −6 μV to 10 μV, respectively. The P300 amplitude presents a decreased trend, and the P300 latency presents an extended trend from C1 to C5 for every subject.

### 3.2. P300 Amplitude under Five Levels of Weak Hidden Conditions

In this study, the P300 amplitude was defined as the maximum amplitude from 200 ms to 900 ms (the time point at which the stimuli emerged was set at 0 ms). This is because of the specific phenomenon of the maximal positive component being prolonged in the ERP wave morphology (shown in Figure 5) under these proposed paradigms compared with the typical P300 wave of the event-related potential [43]. Table 3 shows the ground average P300 amplitude for all subjects under five conditions. From the table, we can see that the amplitude varies under five conditions. The averaged amplitudes of all subjects under five conditions were 8.92 ± 1.24 μV, 8.55 ± 1.27 μV, 8.51 ± 1.32 μV, 8.72 ± 1.33 μV, and 7.84 ± 1.40 μV. We conducted a paired *t*-test between benchmark condition C1 and other conditions to assess the statistically significant differences. Although the averaged amplitude presents a decreasing trend from C1 to C5, the averaged amplitude decreased significantly only under C5, compared with C1. Hereafter, “*” indicates a significant difference unless noted: “*”, “**”, and “***” represent *p* < 0.05, *p* < 0.01, and *p* < 0.001, respectively.

### 3.3. P300 Latency under Five Levels of Weak Hidden Conditions

The P300 latency was defined as the corresponding time point to the P300 amplitude. Table 3 shows the ground average P300 latency for all subjects under five conditions and the significant difference between C1 and other conditions. From the table, we can see that the latency also varies between subjects given a specific condition, similar to the amplitude. The averaged latencies of all subjects under five conditions were 582.39 ± 25.02 ms, 572.44 ± 25.72 ms, 575.83 ± 23.57 ms, 595.00 ± 21.15 ms, and 643.83 ± 26.16 ms. We conducted a paired *t*-test between C1 and the other conditions to assess the statistically significant differences in a similar way to the P300 amplitude. Different from the average amplitude, the average latency presented a U-shaped trend instead of a monotonical trend, similar to the P300 amplitude. More narrowly, the average latency shortened from condition C1 to C2 and extended from C2 to C5. Specifically, the shortest average latencies were obtained under conditions C2 and C3. Despite the above results for all subjects under five conditions, the average latency only changed significantly under condition C5, compared with condition C1.

### 3.4. Channel Selection under Five Levels of Weak Hidden Conditions

**Figure 6:**
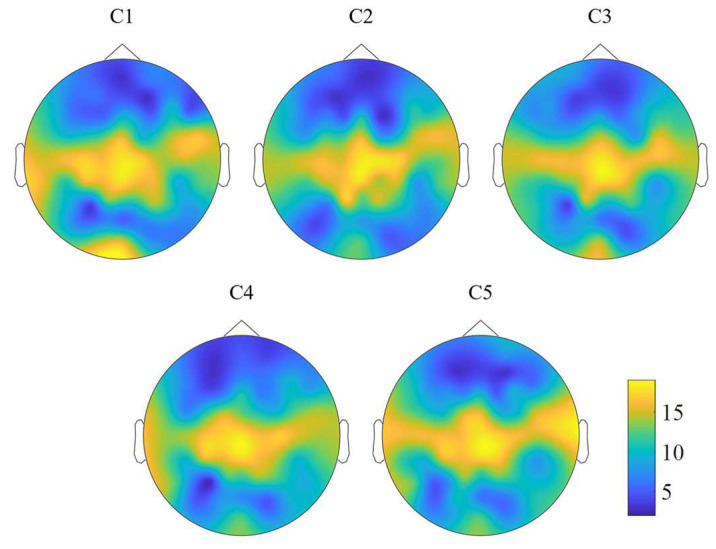
Distribution of the number of selected channels of all subjects under five levels of weak hidden conditions.

Channel sets were selected to reduce the channel number, system setup cost, and time. We counted the number of selected channels for all subjects under five conditions, as shown in Figure 6. From the figure, we can see that although the distribution of the number of selected channels differed between each condition, the main selected areas were the same. For each condition, most channels were selected from the parietal lobe of all subjects. Many channels were also selected from the occipital lobe for condition C1 and the right temporal lobe for condition C5, which is consistent with the results of previous studies [44,45]. Some channels were also selected from the left and right motor areas.

### 3.5. Performance under Five Levels of Weak Hidden Conditions and Six Channel Sets

Recognition performance was evaluated by the decoding algorithms with different channel sets for each subject under each condition. Table 4 shows the detection performance (mean with standard error) of six channel sets under five conditions by LDA and SVM classifiers and the significant difference between C1 and other conditions under the same channel set. From the table, we can see that the decoding performance presents a decreasing trend from condition C1 to C5 for each set, both for LDA and SVM. However, the classification accuracy did not decrease by more than 5% from condition C1 to C5 for each set, using LDA and SVM, as the channel number of the channel set decreased from 62 to 6. Comparable recognition performance between LDA and SVM was obtained. The best performance was obtained using channels of channel set 1 under condition C1, both for the LDA and SVM classifiers, with an accuracy of 79.65 ± 2.92% and 78.94 ± 2.83%, respectively. For the LDA classifier, the worst performance was obtained using the channels of channel set 6 with an accuracy of 72.62 ± 3.14%, while the worst performance for the SVM classifier was obtained using the channels of channel set 5 with an accuracy of 73.27 ± 3.14%. The interaction effect between weak hidden condition and channel set was investigated using a two-way repeated measures ANOVA. The results showed that the interaction effect between weak hidden condition and channel set was not significant for either the LDA classifier (*F*(20, 510) = 0.029, *p* > 0.05) or SVM classifier (*F*(20, 510) = 0.018, *p* > 0.05).

The information transfer rate is shown in Table 5. From the table, we can see that the information transfer rate was higher than 50 bits/min for all channel sets under the five conditions using the LDA and SVM classifiers. The minimum information transfer rate was 50.07 ± 4.21 bits/min for channel set 6 under condition C5 using the LDA classifier. The highest information transfer rate was 60.37 ± 4.34 bits/min for channel set 1 under condition C1 using the LDA classifier. The information transfer rate presented a similar trend to the classification accuracy.

A paired *t*-test was conducted between C1 and the other conditions to assess the statistically significant differences. The performance changed significantly between C1 and C5 for all channel sets by the LDA and SVM classifiers, while other channel sets did not. With the fewest channels (i.e., channel set 6) under conditions C1 and C5, the classification performance changed significantly from 75.20 ± 2.80% to 72.62 ± 3.14% using the LDA classifier and from 75.56 ± 2.89% to 73.35 ± 3.15% using the SVM classifier; the classification accuracy reductions were both less than 3%.

**Figure 7:**
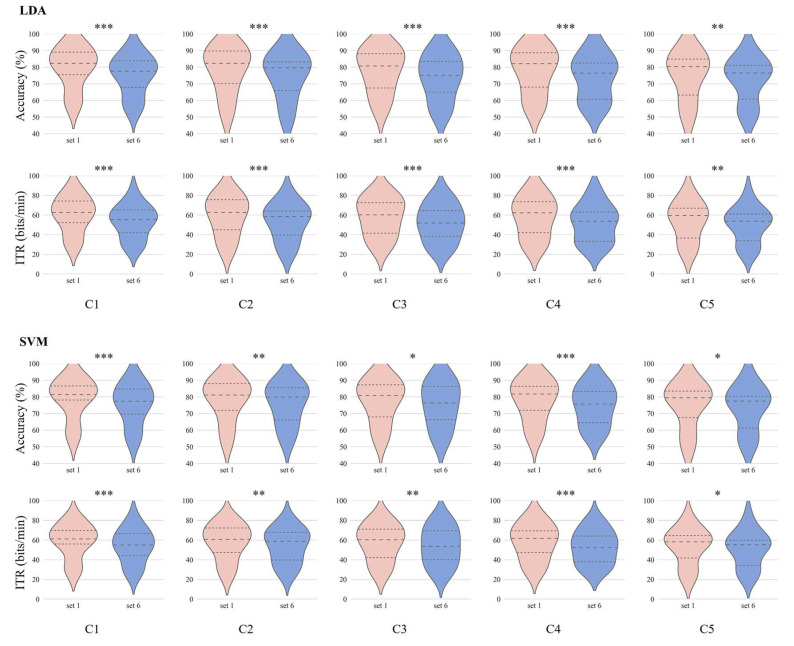
Violin charts of the classification accuracy and information transfer rate of channel set 1 and set 6 under five levels of weak hidden conditions. *, **, and *** represent *p* < 0.05, *p* < 0.01 and *p* < 0.001, respectively.

Figure 7 shows the violin charts of the classification accuracy and information transfer rate of channel sets 1 and 6 for all subjects under five conditions. In addition, it shows the significant difference between channel sets 1 and 6 for all subjects under five conditions using the LDA and SVM classifiers. From the figure, we can see that although the classification accuracy and information transfer rate changed significantly between sets 1 and 6 under five conditions, both for the LDA and SVM classifiers, the median accuracy and information transfer rate decreased slightly.

## 4. Discussion

In this study, we defined the weak hidden conditions quantitatively based on the RGB color space and designed RSVP paradigms corresponding to five levels of weak hidden conditions. Neural signatures, including P300 latency and amplitude under five levels of weak hidden conditions, were explored. The optimal channel sets were determined by the channel selection method for each subject under each weak hidden condition. Then, detection performance, including classification accuracy and the information transfer rate, was investigated.

The P300 amplitude presented a decreasing trend from condition C1 to C5, while the latency first decreased from condition C1 to C2 and then increased from C2 to C5. A previous study demonstrated that when a subject’s attention is directed away from the task or stimulus, P300 amplitude decreases [46]. Consequently, a reason for the P300 amplitude change phenomenon may be that the subject missed the target stimuli in some trials because of the high presentation speed and increasing fatigue during the experiment. It is likely to at least be true for some subjects and the ERP was not elicited in some trials. Another reason may be that it is really the level of a weak hidden condition that significantly influences the P300 amplitude with a positive correlation, according to the findings of the authors in [47]. This is despite the insignificant results based on the P300 speller developed by Farewell and Donchin in [48]. For the P300 latency change phenomenon, increasing attention should intuitively be paid to quickly recognizing the target stimuli from conditions C1 to C5. P300 latency will be prolonged when the categorization of the stimulus becomes more difficult, and this reflects a longer duration of mental processes [49,50]. For the above results, one possible reason may be that increased attention did not add too much value, i.e., it was easy to recognize the target stimuli from condition C1 to C4, but the difficulty level then sharply increased, i.e., it was difficult to recognize and categorize the target under condition C5. Although the target was difficult to recognize at first sight under C5, according to the subjects’ feedback, the P300 potential was elicited by the target successfully. One possible reason may be that although the stimuli of the two RSVP paradigms in successive trials may significantly change, especially from condition C1 to C5 directly, the subjects’ eyes could quickly become used to the weak hidden condition from the previous trial to the current trial when the current plus sign occurred. Thus, the subject could perceive the target stimulus, and the ERP wave was elicited successfully during the stimuli presentation.

The classification performance was investigated for all subjects under five conditions using LDA and SVM classifiers. The classification performance showed a similar trend with the P300 amplitude under five conditions for the same channel set and same classifier despite the different trend with the P300 latency. This indicated that a higher P300 amplitude would lead to better classification performance [51]. The performance did not differ greatly between LDA and SVM with the same experimental factors (e.g., classification feature, channel number, and condition). The best performance was observed under condition C1. For each channel set, although the classification accuracy showed a decreasing trend from condition C1 to C5, the accuracy reductions were less than 5%. Specifically, the accuracy reductions were less than 3% for channel set 6 both for LDA and SVM from conditions C1 to C5.

For each condition, the channels from the parietal lobe were selected frequently from the subject, which reflected the vast difference between the brain patterns in the parietal lobe elicited by target stimuli and non-target stimuli. Many channels were also selected from the occipital lobe for condition C1. One possible reason for this phenomenon may be that the stimuli for condition C1 were the most conspicuous, which the subjects were sensitive to, and they could easily activate the subjects’ visual area. From channel set 1 to channel set 6, the classification accuracy decreased by less than 5%. Compared with channel set 1, i.e., the total channels, the classification accuracy only decreased by less than 2% (from 75.04% to 73.35%) with the SVM classifier under condition C5. In contrast, the channel number decreased by over 90%, which may sharply reduce the system setup time and cost.

There are some limitations of this study. This study only focused on the weak hidden conditions related to the grayscale pattern targets. Nevertheless, there are also other types of weak hidden conditions pertaining to factors such as the target category (e.g., letters, human faces, human contours, medical images, and aerial-related images), target size, target shape, target color (e.g., pure or complex), the distance between the subject and the target, and so on. Targets that are less interesting and those that are a smaller size, a more similar shape, a more similar color, and at a further distance will result in a higher level of weak hidden conditions, which is worthwhile to investigate. Furthermore, pure and monotonous backgrounds were investigated in this study, while complex and cluttered backgrounds should also be taken into consideration.

Environmental illumination is another factor. In this study, we only experimented in a room lit by fluorescent lamps to simulate daytime environment conditions. But, in the experiment setup, we found that the recognizability (human visual perception) of the targets under weak hidden conditions can be influenced by environmental illumination. The subjects could hardly recognize the number in conditions C4 and C5 when the fluorescent lamp was off during the experiment at a distance of 50 cm. High environmental illumination is helpful for target detection under weak hidden conditions, while low environmental illumination is not. This difference will probably result in different detection performances.

The classification performance is perhaps not high enough for efficient detection in practice. Other channel selection methods [39,52], machine learning algorithms, and deep learning neural networks, such as EEGNet [53], spatial–temporal neural networks [54], and other deep neural networks [31,32,55,56], can be explored to improve the recognition performance for practical applications.

## 5. Conclusions

In this study, we proposed rapid serial visual presentation (RSVP) paradigms for target detection corresponding to five levels of weak hidden conditions quantitively based on the RGB color space. Eighteen subjects participated in the experiment, and neural signatures, including P300 amplitude and latency, were investigated. Detection performance was evaluated under five levels of weak hidden conditions using the linear discrimination analysis and support vector machine classifiers on different channel sets. The experimental results showed that, compared with the benchmark condition, (1) the P300 amplitude decreased and latency was prolonged significantly only under the weakest hidden condition, and (2) the detection accuracy decreased by less than 2% with more than a 90% reduction in channel number (62 channels versus 6 channels), determined using the proposed channel selection method under the weakest hidden condition. Our study can provide new insights into target detection under weak hidden conditions based on EEG signals with a rapid serial visual presentation paradigm, and it may expand the application of brain–computer interfaces in EEG-based target detection areas.

Our future work aims to improve recognition performance using deep learning methods; explore neural signatures under other types of weak hidden conditions, including different stimulus sizes, shapes, environmental conditions, and cluttered backgrounds; and explore the effects of environmental illumination on neural signatures and recognition performance.

## Figures and Tables

**Table 1:** RGB of the stimuli and the background of the RSVP paradigms under five levels of weak hidden conditions.

RGB	Background	Condition				
		C1	C2	C3	C4	C5
R	0	255	127	63	31	15
G	0	255	127	63	31	15
B	0	255	127	63	31	15
Ratio with C1	0	1	0.498	0.247	0.122	0.059

**Table 2:** Detailed information regarding the six channel sets.

Channel Set	Channel Number	Channels
1	62	All Channels
2 [38]	32	FP1, FP2, Fz, F3, F4, F7, F8, FC1, FC2, FC5, FC6, Cz, C3, C4, T7, T8, CPz, CP3, CP4, Pz, P3, P4, P7, P8, POz, PO3, PO4, PO7, PO8, Oz, O1, O2
3 [39]	16	Fz, FC1, FC2, Cz, C3, C4, CP1, CP2, Pz, P3, P4, P7, P8, Oz, O1, O2
4	8	FPz, Fz, FCz, Cz, CPz, Pz, POz, Oz
5 [38]	8	Fz, Cz, Pz, P3, P4, PO7, PO8, Oz
6	6	Selected channels

**Table 3:** Ground average P300 amplitude (μV) and latency (ms) of all subjects under five levels of weak hidden conditions.

Condition	Amplitude (μV)	Latency (ms)
C1	8.92 ± 1.24	582.39 ± 25.02
C2	8.55 ± 1.27	572.44 ± 25.72
C3	8.51 ± 1.32	575.83 ± 23.57
C4	8.72 ± 1.33	595.00 ± 21.15
C5	7.84 ± 1.40 *	643.83 ± 26.16 *

“*” indicates a significant difference between C1 and other conditions: * represents *p* < 0.05.

**Table 4:** Classification accuracy (mean ± standard error) for the LDA and SVM classifiers using all channel sets under five levels of weak hidden conditions (%).

Set	LDA					SVM				
	C1	C2	C3	C4	C5	C1	C2	C3	C4	C5
1	79.65 ±2.92	78.27 ±3.33 *	77.81 ±3.13 *	77.66 ±3.29 *	74.96 ±3.42 ***	78.94 ±2.83	78.05 ±3.00	77.49 ±3.02	78.07 ±2.96	75.04 ±3.24 **
2	79.24 ±2.99	78.62 ±3.15	77.02 ±3.13 *	77.73 ±3.20	74.45 ±3.36 ***	78.70 ±2.89	78.13 ±2.86	77.11 ±3.01	77.71 ±2.85	74.85 ±3.16 **
3	77.82 ±2.95	77.56 ±3.18	76.21 ±3.14	75.05 ±3.29 **	73.67 ±3.28 **	78.04 ±2.81	77.36 ±2.83	77.02 ±2.92	76.08 ±2.96 *	73.81 ±3.05 ***
4	78.15 ±3.03	77.09 ±3.26 *	76.46 ±3.16	77.20 ±2.99	74.36 ±3.32 *	77.30 ±2.86	77.07 ±2.85	76.54 ±2.99	76.94 ±2.95	73.82 ±3.25 *
5	78.05 ±2.98	78.25 ±3.23	76.34 ±3.16	75.78 ±3.20 **	73.41 ±3.17 ***	77.73 ±2.78	77.02 ±2.97	76.59 ±3.03	76.20 ±2.88 *	73.27 ±3.10 ***
6	75.20 ±2.80	74.64 ±3.28	73.90 ±3.19	73.63 ±3.15	72.62 ±3.14 *	75.56 ±2.89	75.54 ±2.94	75.12 ±3.17	74.64 ±2.81	73.35 ±3.15 *

*, **, and *** represent *p* < 0.05, *p* < 0.01 and *p* < 0.001, respectively.

**Table 5:** Information transfer rate (mean ± standard error) for LDA and SVM classifiers using all channel sets under five levels of weak hidden conditions (bits/min).

Set	LDA					SVM				
	C1	C2	C3	C4	C5	C1	C2	C3	C4	C5
1	60.37 ±4.34	58.70 ±4.78	57.75 ±4.57 *	57.78 ±4.82 *	53.79 ±4.68 ***	59.05 ±4.05	57.88 ±4.24	57.09 ±4.33	57.93 ±4.32	53.62 ±4.36 ***
2	59.82 ±4.44	59.02 ±4.57	56.56 ±4.54 *	57.76 ±4.70	52.96 ±4.56 ***	58.75 ±4.13	57.84 ±4.07	56.49 ±4.29	57.26 ±4.19	53.27 ±4.30 **
3	57.51 ±4.26	57.39 ±4.53	55.35 ±4.55	53.81 ±4.64 **	51.73 ±4.45 **	57.65 ±4.03	56.63 ±4.03	56.24 ±4.18	54.87 ±4.20 *	51.61 ±4.06 ***
4	58.12 ±4.37	56.77 ±4.57 *	55.73 ±4.50 *	56.64 ±4.34	52.79 ±4.54 **	56.59 ±4.06	56.21 ±4.03	55.64 ±4.29	56.18 ±4.26	51.90 ±4.38 **
5	57.93 ±4.34	58.52 ±4.61	55.53 ±4.51	54.74 ±4.51 **	51.22 ±4.28 ***	57.15 ±4.01	56.25 ±4.16	55.73 ±4.33	54.97 ±4.10 *	50.90 ±4.11 ***
6	53.40 ±3.94	53.12 ±4.47	52.00 ±4.46	51.60 ±4.47	50.07 ±4.21 *	54.04 ±4.08	54.05 ±4.12	53.76 ±4.52	52.62 ±4.00	51.14 ±4.31 *

*, **, and *** represent *p* < 0.05, *p* < 0.01 and *p* < 0.001, respectively.

## Data Availability

The data included in this study are available upon reasonable request by contacting the corresponding author.
